# Franks and Farquhar (2007) and beyond: the influence of guard and subsidiary cell biomechanics on stomatal function

**DOI:** 10.1093/plphys/kiag724

**Published:** 2026-10-10

**Authors:** Tracy Lawson, Michelle Facette, Scott A M McAdam, Tanja A Hofmann

**Affiliations:** Department of Plant Biology, University of Illinois at Urbana-Champaign, 286 Morrill Hall, 505 South Goodwin Avenue, Urbana, IL 61801, United States; Carl R Woese Institute for Genomic Biology, University of Illinois at Urbana-Champaign, Urbana, IL 61801, United States; Department of Crop Sciences, University of Illinois at Urbana-Champaign, Urbana, IL 61801, United States; School of Life Sciences, University of Essex, Wivenhoe Park, Colchester CO4 3SQ, United Kingdom; Department of Biology, University of Massachusetts Amherst, Amherst, MA, United States; Department of Botany and Plant Pathology, Purdue University, 915 Mitch Daniels Blvd, West Lafayette, IN 47907, United States; Department of Plant Biology, University of Illinois at Urbana-Champaign, 286 Morrill Hall, 505 South Goodwin Avenue, Urbana, IL 61801, United States

## Abstract

Stomata are the gatekeepers to leaf gas exchange and as such are essential for CO_2_ uptake for photosynthesis, water loss for evaporative cooling, and ultimately plant growth and productivity. Research has long focused on understanding the mechanisms that govern stomatal aperture adjustments, in particular how changes in turgor pressure in guard cells (GCs) are achieved and how these cause the reversible cell shape distortion that leads to pore opening and closing. Here we discuss the immense impact the paper by Franks and Farquhar (2007) has had on this research field, both in the past and more recently, by stimulating new angles of exploration of stomatal mechanics. As new techniques have emerged, the impact of the mechanical advantage of epidermal/subsidiary cells and how this is overcome by GCs has inspired novel studies into the importance of subsidiary to stomatal function, the effect of different cell shapes (dumbbell- vs kidney-shaped) on stomatal kinetics, the significance of cell geometries and cell complexes, as well as the underlying biochemical cell wall composition responsible for GC deformation. While many questions remain unanswered, we summarize the take-home messages from the key literature on stomatal biomechanics.

## Introduction

The adjustable stomatal pores on leaves are the gatekeepers to gaseous diffusion between the atmosphere and the leaf interior being critical to CO_2_ uptake to meet the demands for photosynthesis. Stomatal opening comes at the cost of water loss, which is an order of magnitude greater than CO_2_ diffusion, but critical for nutrient uptake and evaporative cooling (Matthews et al. 2017). The guard cells (GCs) that surround the pore alter in turgor to change aperture, driven by internal and external environmental cues to increase or decrease the flux of gases. In general, opening is driven by increasing or high light intensity (through at least 2 well-established signaling pathways (Pelech et al. 2026) and low CO_2_ concentration [CO_2_], while closure is driven by the reverse, as well as low humidity and reduced plant water status (although the exact mechanism(s) of how stomata respond to environmental factors that influence plant water status are still not entirely clear (Buckley 2019), see below). The rate of gaseous exchange depends on stomatal aperture and density (or number of stomata per unit area) and is typically measured as stomatal conductance (*g_s_*), defined as the maximum capacity for gaseous diffusion (usually measured as water flux). Stomatal aperture is a function of both GC turgor pressure and GC morphology (Lawson and Blatt 2014; Lawson and Matthews 2020; Burgess and Ugalde 2024). More than a century of research has identified different morphologies and has elucidated many of the signal transduction pathways, receptors, and osmoregulatory processes in these cells that are required for optimal functioning. Although stomatal morphology has several common characteristics, it can also vary greatly between species and plant types (Matthews et al. 2017). The stomatal pore is always surrounded by two GCs; however, these differ in shape, with two categories that are distinct in structure and form, ie kidney or elliptical shaped and dumbbell or graminoid type. Dumbbell-shaped GCs are found in grasses, with relatives from the order Poales also having non-oblique paracytic subsidiary cells and varying degrees of guard cell narrowing (Menezes et al. 2025; Petrone-Mendoza 2026, 2026; McAdam et al. 2026), while kidney-shaped GCs are evolutionary much older and most common. Dumbbell-shaped GCs are *always* flanked by paracytic GC subsidiary cells, and together with the GCs they make up the stomatal complex (Lawson and Matthews 2020). Subsidiary cell (SCs) are cells surrounding the GCs defined as morphologically distinct from other epidermal cells (Rudall et al. 2013, 2017; Rudall and Knowles 2013). In species with kidney-shaped GCs, these SCs may or may not be present and depend on the species. The number and shape of SCs varies greatly (Metcalfe and Chalk 1950; Rudall et al. 2013, 2017; Rudall and Knowles 2013; Gray et al. 2020; Lawson and Matthews 2020), with reports of up to 10 SCs within a complex (Metcalfe and Chalk 1950). Although it has been recognized that these cells play an important role in stomatal function (Franks and Farquhar 2007; Raissig et al. 2017), the majority of studies have focused on the paracytic SCs adjacent to the GCs, with the role of secondary lateral and terminal subsidiary cells not entirely clear. Some of the earliest studies in stomatal structure and function were by Hugo von Mohl (1856) and he was one of the first to recognize an antagonism between GCs and epidermal cells, and that high epidermal turgor had a closing influence on stomata. Subsequently several authors reported that epidermal cell turgor has a “mechanical advantage” over the GCs and this must be overcome for stomata to open (DeMichele and Sharpe 1973; Cooke et al. 1976; Wu et al. 1985; Franks et al. 1998)

The relationship between GCs and the surrounding subsidiary or epidermal cells was revisited in the *Plant Physiology* paper by Franks and Farquhar (2007) who utilized modern tools to focus on the mechanical diversity of stomata and the impacts on gas exchange. While previous work had elucidated a relationship between the two cell types, little was known about the mechanical characteristics and the impact on performance, and even fewer studies had quantified any turgor differences between GCs and the surrounding epidermal or subsidiary cells in relation to stomatal function. Furthermore, all of the previous studies had focused on kidney-shaped GCs, with no information available on dumbbell-shaped GCs. The authors addressed this oversight by coupling two technically challenging methods, cell pressure probes to quantify GC and epidermal/subsidiary cell turgor pressure and cryo-SEM to visualize stomatal aperture of four different species with distinct morphologies and evolutionary history. Three species selected had kidney-shaped GCs: (i) a lycophyte *Huperzia prolifera*, sister to all other vascular plants, with large GCs lacking SCs (anomocytic); (ii) a fern *Nephrolepsis exaltata;* which has comparable stomatal dimensions to the lycopod and is also anomocytic, but achieves greater apertures and has higher photosynthetic rates; and (iii) a herbaceous angiosperm, *Tradescantia virginiana* which has paracytic and polar (apical and lateral) SCs and high photosynthetic rates. The fourth species was the grass *Triticum aestivum*, which has dumbbell-shaped GCs with paracytic SCs, typical of all grasses. This unique GC complex has been linked to rapid stomatal movement in grasses and been suggested to be a key to the global success of grasses in terms of dispersal (Brench et al. 2026).

Using impressive cryo-scanning electron micrographs (Fig. 1) the authors illustrated the “signature GC swelling and displacement characteristics” of the species studied. In the lycopod and fern species limited mechanical interaction between the GCs and surrounding epidermal cells was evident. The authors demonstrated that during stomatal opening of the species with distinct SCs (*Tradescantia* and *Triticum*) GC swelling led to “lateral displacement into the adjacent SCs” and the SCs had to “accommodate” this. However, no such interactions between the two cell types were observed in the lycopod or fern species (with no SCs), which relied entirely on GC swelling resulting in GC shape changes allowing opening without any impact on surrounding epidermal cells. Critical to this experiment was that water potential for all cells was close to zero as samples were collected at a very high humidity. By using this approach, the authors demonstrated that for *Tradescantia* and *Triticum* to achieve the maximum recorded aperture, the turgor pressure of the surrounding SCs had to greatly reduce. This was only possible by a substantial reduction in osmotic pressure in SCs, which enables GCs to overcome the mechanical constraint imposed by them and expand into SC space (Fig. 1). Turgor pressure measurements of the guard and surrounding subsidiary or epidermal cells showed that there was a linear relationship between GC turgor pressure and aperture. This relationship was greatly affected by SC pressure in *Tradescantia* and *Triticum* with a high mechanical advantage but completely unaffected by epidermal cell pressure in the other two. These measurements of stomatal aperture in conjunction with subsidiary/epidermal and GC turgor provided the foundational empirical data for models of stomatal function (Buckley 2019).

**Figure 1:**
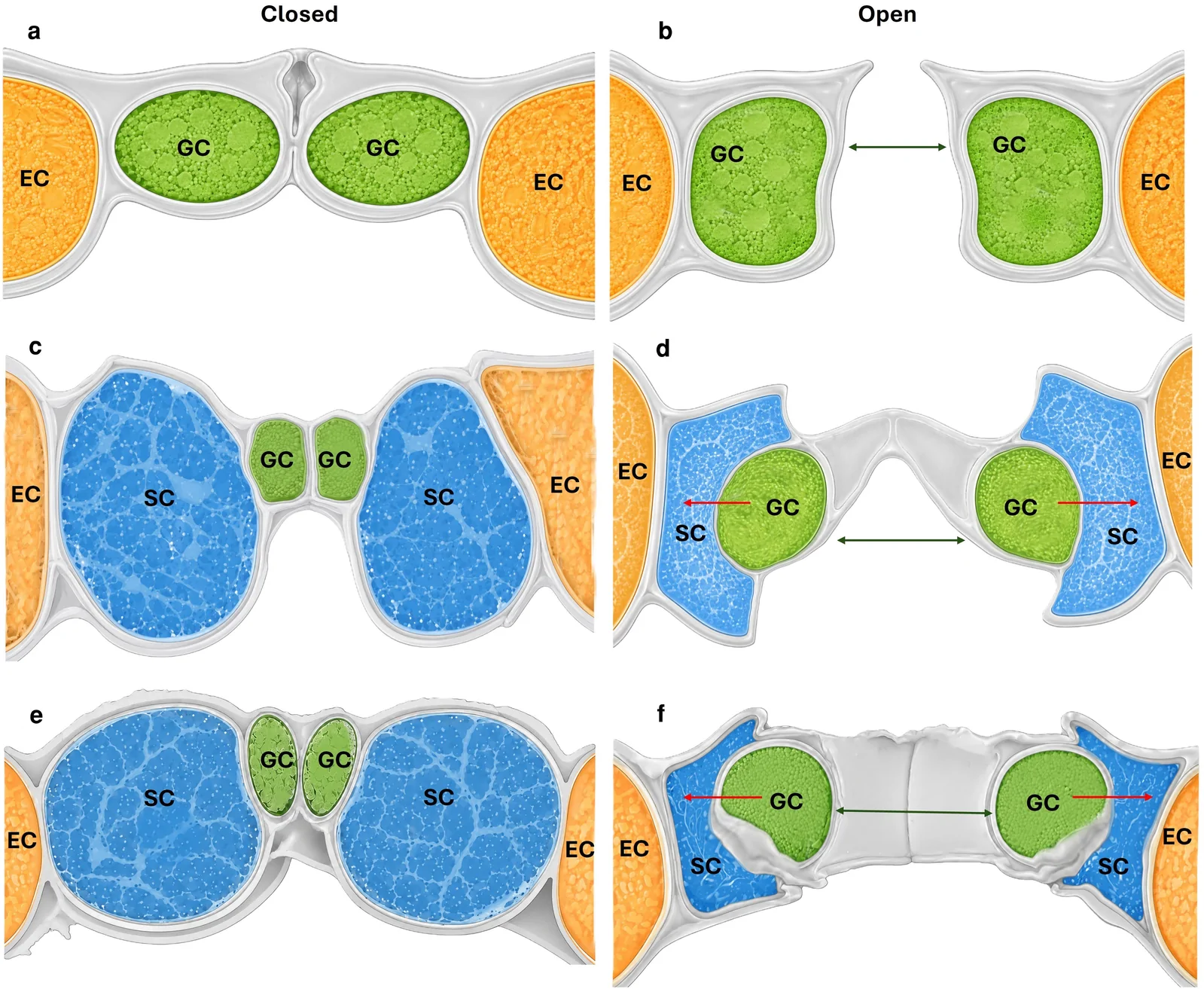
Line schematics redrawn from original Figs. 2–5 in Franks and Farquhar (2007) showing cross sections of open and close stomata from *H. prolifera* (a & b); *T. virginiana* (c & d); *T. aestivum* (e & f). EC denotes epidermal cells; GC guard cells and SC subsidiary cells position. Red arrows demonstrate the increase in GC size during open occupying SC space. Green arrows illustrate the pore opening. Illustrations were generated using OpenAI ChatGPT image generation based on annotated scanning electron microscopy (SEM) images. The resulting images were manually edited and refined in Adobe Photoshop 27.7.0 (Adobe Inc., San Jose, CA, USA). Shellie Wall is acknowledged for generating the Figure.

Earlier studies reported that SCs act as a reservoir for solutes for GC osmoregulation, and they quantified the antagonistic relationship in terms of units of turgor pressure between GCs and SCs (Edwards and Meidner 1975; Meidner and Edwards 1975; Edwards et al. 1976). However, the discovery of this important osmotic relationship must be attributed to Raschke and colleagues (Raschke and Fellows 1971; Raschke 1976; Raschke and Schnabl 1978), who were the first to show K and Cl ions shuttling between SCs and GCs in the paracytic maize stomatal complex (Raschke and Fellows 1971). Franks and Farquhar (2007) built upon these findings using a modeling approach that combined osmoregulation and cell mechanics, to demonstrate the “see-sawing” mechanism of solute transfer (mostly K^+^) that results in a swap of osmotic and turgor pressure between the 2 cell types. Their work highlighted the “mandatory and opposite” nature of this mechanism in which both solutes and water (and therefore turgor) move between the subsidiary/epidermal cells and the GCs. The process appears obligate in species with a large mechanical advantage to achieve maximum aperture and is diminished in species with reduced mechanical advantage. The benefit of this mechanism is not only an increased stomatal aperture but also linked to more rapid stomatal kinetics supporting increased rates of photosynthesis and water use efficiency and may have been an important aspect in the evolution of stomatal function. Franks and Farquhar (2007) concluded that the rapid stomatal responses in species with dumbbell-shaped GCs (with their associated SCs) was driven by a combination of osmotic shuttling, a high mechanical advantage, and distinct GC geometry. Subsequent studies (Jezek et al. 2017; Wang et al. 2017; Pichaco et al. 2024) have shown that the contribution of turgor from surrounding epidermal cells needs to be integrated into models of GC dynamics for stomatal kinetics to be accurately (Wang et al. 2017) predicted.

The *Plant Physiology* paper by Franks and Farquhar (2007) is considered a seminal manuscript, as it has reframed our understanding of what controls stomatal function and directed subsequent research in the area to include mechanical diversity, and the impacts the distinct differences in anatomy can have on behavior. This has also been key in research focusing on the evolutionary tuning of stomatal form and function and has spurred and contributed to several topical areas of stomatal research including: (i) the importance of stomatal kinetics for breeding for crops with improved photosynthesis and water use efficiency; (ii) the importance of GC geometry and cell wall properties in stomatal function; and (iii) probably most impactfully, it has helped to drive an entire research area that focuses on SCs within the stomatal complex, their importance in structure, as well as the emerging new fields that aim to understand the osmoregulation and communication between SC and GC. Here we will briefly introduce and highlight the latest findings in these areas.

## Evolutionary tuning of stomatal form and function

The seminal discovery of Franks and Farquhar (2007), that there were major differences in the mechanical regulation of stomatal aperture across land plant clades, is now broadly recognized (Brodribb and McAdam 2011; Gong et al. 2021), and along with the work of Doi and Shimazaki (2008) in *Plant Physiology* the following year on stomatal responses to light and CO_2_, has provided a catalyst for now two decades of research characterizing the evolutionary diversity of stomatal function in land plants (Sussmilch et al. 2019a). Evolution in how stomatal aperture is regulated by leaf water status, first described by Franks and Farquhar (2007), has played a central role in shaping the past 400 million years of vascular plant diversification on land (Brodribb and McAdam 2011; Gong et al. 2021; McAdam and Sussmilch 2021).

Research inspired by the discovery of Franks and Farquhar (2007), has found that across lycophytes, most ferns and gymnosperms epidermal turgor does not influence stomatal aperture—unlike in angiosperms (Martins et al. 2016; Deans et al. 2017; Gong et al. 2021; Suissa et al. 2023). Consequently, in the absence of mechanical interactions, GC turgor and stomatal aperture will change in unison with changes in leaf water status in non-angiosperms (Brodribb and McAdam 2011; McAdam and Brodribb 2012; Gong et al. 2021). This “passive” response relies on the simple swelling and shrinking of GCs depending on leaf water potential only (Buckley 2019). Therefore, this means stomatal responses to changes in leaf water status are highly predictable in these species (Brodribb and McAdam 2011; Cardoso et al. 2019).

The evolution of a mechanical link between GCs and neighboring subsidiary/epidermal cells in angiosperms dramatically alters how stomatal responses to changes in leaf water status are controlled (Buckley 2019). While capitalizing on an increased aperture due to mechanical interactions (Rockwell and Holbrook 2017), when angiosperms are exposed to high vapor pressure deficit (VPD), which drives stomatal closure, stomata initially experience a “wrong-way response,” transiently increasing aperture before actively closing. This is due to a loss of epidermal cell turgor (Buckley et al. 2011; Binstock et al. 2024) and reduction in epidermal pressure forcing GCs closed. Consequently, angiosperms cannot rely solely on passive changes in GC turgor to regulate transpiration when leaf water status changes. Franks and Farquhar (2007) hypothesized that a “highly active compensating mechanism” was required in angiosperms to overcome the effect of low epidermal turgor and to reduce stomatal aperture at high VPD. It has since been revealed that the hormone abscisic acid (ABA), which is rapidly synthesized in angiosperm leaves at high VPD, provides this closing mechanism by actively lowering GC turgor (Bauer et al. 2013; McAdam et al. 2016; Jalakas et al. 2021). Although recently in *Arabidopsis*, Wang et al. (2017) demonstrated that ABA is not an essential factor in determining stomatal responses to VPD, and that some change in stomatal aperture can be driven by alterations to apoplastic water potential as well (Blatt et al. 2026). Working on the evolution of stomatal responses to ABA across land plant lineages, McAdam and Brodribb (2012) showed that the stomata of lycophytes and ferns are insensitive to endogenous ABA synthesized under drought, and suggested that major evolutionary transitions in the molecular signaling pathways for the control of stomata by ABA occurred in the ancestor of the seed plants and angiosperms (Sussmilch et al. 2019a, 2019b; Sussmilch et al. 2024), (although Cai et al (2017) have reported that ABA sensitivity to exogenous applications is present in some fern lineages). This difference in stomatal regulation by water status across land plant lineages, first recognized by Franks and Farquhar (2007), has been suggested to have had profound ecological and evolutionary consequences (Sussmilch et al. 2019a; McAdam et al. 2021). Modern lycophytes and ferns, unless able to survive from a large capacitance or complete desiccation, are ecologically confined by a passive stomatal response to changes in leaf water status (McAdam and Brodribb 2013; Prats and Brodersen 2021; Prats et al. 2024). In contrast, angiosperms evolved greater rates of gas exchange by having GCs that can open by displacing neighboring epidermal cells (Franks and Farquhar 2007), as well as an active means of regulating stomatal aperture in response to rapid changes in leaf water status (Brodribb and McAdam 2017). This provided a novel phenotype, combining both highly productive leaves with an ability to survive periods of low water status with efficiently closed stomata (Brodribb et al. 2007; Westbrook and McAdam 2021) which may have allowed angiosperms to rapidly dominate terrestrial ecosystems.

Although it has been suggested that an active (hydroactive) response in lycophytes and ferns is not needed because these plants lack the mechanical advantage of the epidermal cells (Franks and Farquhar 2007; Buckley 2019), the lack of a stomata response to ABA in seed-free plants remains heavily debated. Several reports have shown that the genes required for ABA biosynthesis and signaling pathway *are* present in ferns and some mosses (Chater et al. 2011; Lind et al. 2015; Cai et al. 2017), and that exogenously applied ABA can close stomata in some seed-free plants (Ruszala et al. 2011; Hõrak et al. 2017). Deletion of OST1 (a gene involved in ABA responses) ortholog in the moss *Physcomitrium* resulted in impairments in stomatal closure, suggesting possible active mechanisms (Chater et al. 2011), although recent work demonstrating that bryophyte stomata are not turgor-operated valves challenges this (Brodersen et al. 2025). Stomata aperture in species from early diverging lineages is generally much lower than in angiosperms, due to a lower photosynthetic demand (McElwain et al. 2016) which might be linked to a more reduced sensitivity to active signals. Even after nearly two decades since the work of Franks and Farquhar (2007), further research is ongoing in this highly debated area of research to fully elucidate the evolution of hydropassive and hydroactive mechanisms across lineages and plant functional types.

## Speed of stomatal responses

Franks and Farquhar (2007) also highlighted that differences in GC morphologies not only influence overall stomatal aperture but also have implications for the speed of stomatal responses, which encouraged research in this area. Initial studies linked the speed of stomatal responses to the size of the stomata, with small stomata responding more quickly (Hetherington and Woodward 2003; Franks and Farquhar 2007; Franks and Beerling 2009; Drake et al. 2013; Raven 2014). The greater surface area to volume ratio was proposed to enable enhanced rapidity in smaller stomata compared with larger GCs (Hetherington and Woodward 2003). However, in later studies, size could not explain some of the differences reported (Elliott-Kingston et al. 2016; McAusland et al. 2016); recently Woning et al. (2026) used data from 89 different species and confirmed that size–speed relationship is not universal and depends on “species-specific traits, stomatal type and broader anatomical and physiological features.” Interestingly Rui et al. (2024) explored the relationship between the size of guard and SCs and the impact on stomatal kinetics in various C3 and C4 crops under different watering conditions and showed that the GC/SC size ratio significantly impact stomatal kinetics in C4 *Poaceae* crops, but not C3 species.

Early studies examining stomatal responses to various environmental signals, although extensively studied, focused mostly on steady state conditions, with less focus on exploring dynamic stomatal behavior (Lawson and Morison 2004; Lawson et al. 2010). Those that did examine stomatal kinetics did so mostly on understory species and the importance of the speed of stomatal responses on daily carbon gain (Raschke 1970; Kirschbaum et al. 1988; Knapp 1993; Tinoco-Ojanguren and Pearcy 1993; Cardon et al. 1994; Allen and Pearcy 2000; Lawson and Morison 2004; Noe and Giersch 2004). The earlier studies, along with the paper by Franks and Farquhar (2007), led to a flurry of activity in this research area, and in particular, the disconnect between stomatal and photosynthetic responses due to stomata responding an order of magnitude slower than photosynthesis (Lawson et al. 2010, 2012; Lawson and Blatt 2014). As photosynthetic photon flux density (PPFD) is one of the most dynamic natural conditions that affect both photosynthesis and *g_s_*, many studies have used this as stimuli for quantifying stomatal responses (Lawson et al. 2010; Kaiser et al. 2020; Long et al. 2022). Slow stomatal opening in response to increasing light, limits CO_2_ diffusion and photosynthesis by up to 10% (McAusland et al. 2016; Long et al. 2022), which can impact yield (Roche 2015; Adachi et al. 2019), while slow closing can lead to unnecessary water loss after light intensity decreases (Lawson et al. 2012), eroding water use efficiency by up to 20% (Lawson and Blatt 2014), with some reported values in excess of 80% (Papanatsiou et al. 2019; Horaruang et al. 2022). McAusland et al. (2016) was one of the first reports that explored temporal responses in *A* and *g_s_* in relation to carbon assimilation and water use efficiency; they quantified stomatal kinetics in a range of crop and wild species demonstrating species-specific variation in both the speed of *g_s_* and the magnitude of change. They also showed that the majority of species had a greater rapidity in closure than opening suggesting water conservation was an overriding response. These authors confirmed that graminoid stomata were faster (on average 10 min) than elliptical shaped types, due to the see-saw hypothesis of Franks and Farquhar (2007), but also suggested that photosynthetic type (C3/C4) plays a role in these kinetics, which was later confirmed by others (Israel et al. 2022; Ozeki et al. 2022).

Interestingly, Alvim et al. (2024) (building on the work of Horaruang et al. (2022)) suggested rapid stomatal responses in C4 Gynandropsis plants may be due to shifts in the voltage dependence of K^+^ channel gating, which reduced inhibition by extracellular K^+^ and enhanced the capacity for K^+^ fluxes. Excitingly, replacing the Arabidopsis GORK channel with the C4 Gynandropsis homolog resulted in faster stomatal kinetics (Alvim et al. 2025). These findings reinforced the idea that rapidity of stomatal responses, tightly coordinated with mesophyll demands for CO_2_, is a potential novel route to improve both photosynthesis and water-use efficiency, making the speed of *g_s_* changes and the underlying mechanisms that regulate these kinetics a novel breeding target (Lawson and Blatt 2014; McAusland et al. 2016; Fischer and Rebetzke 2018; Faralli et al. 2019).

In general, rapidity of *g_s_* response depends on anatomical feature such as density and size; morphology, cell structure (cytoskeleton and cell wall elasticity) (Carter et al. 2017; Woolfenden et al. 2017), and biochemistry and osmoregulation including the number and activity of ion channels and transporters (Lawson and Vialet-Chabrand 2019; Woning et al. 2026), all of which could provide potential targets for manipulation. For example, Carter et al. (2017) illustrated the importance of pinning down GC ends to increase aperture during opening, while Li et al. (2013) showed that the Actin-Related Protein 2/3 (ARP2/3) complex is important for actin filament remodeling, as well as the fusion of small vacuoles, that facilitates rapid turgor changes required for stomatal opening and closing. Manipulating ion channel activity is another avenue for increasing the rapidity of GC osmoregulation and rapidity. Papanatsiou et al. (2019) used a synthetic blue-light induced K^+^ channel BLINK1 specifically in GCs to increase the flux of K^+^ across the plasma membrane and increased the speed of stomatal opening and closing. These mutants had increased water use efficiency as well as greater biomass. Stomatal kinetics were also improved in Arabidopsis mutants with clustered GC GORK channels that are responsible for K^+^ efflux required for closing, which resulted in reduced water use with no influence on biomass (Horaruang et al. 2022). Other potential targets include co-ordination of tonoplast and plasma membrane transporters (see excellent reviews by Jezek and Blatt 2017 and Nguyen et al. 2023) as well as GC metabolism (Nguyen et al. 2023; Lemonnier and Lawson 2024). It is also worth bearing in mind that considerable natural variation is known to exist within species and breeding populations in response to different environmental stimuli (Faralli et al. 2019, 2022, 2024; Eyland et al. 2021; Pignon et al. 2021; Al-Salman et al. 2023; Battle et al. 2024; Shapira et al. 2024; Yoshiyama et al. 2024; Kumar et al. 2026) providing a wealth of potential targets for crop manipulation. Another interesting area for potential exploitation is ratio of stomata on the adaxial and abaxial leaf surface, as each surface can respond independently and with differences in the *g_s_* kinetics (Wall et al. 2022; Fan et al. 2025). This variation may be due to differences in number and size of stomata as well as the spatial distribution (Shi et al. 2021; Sun et al. 2023; Fan et al. 2025), but also different GC sensitivities to environmental stimuli between the two surfaces (see Wei et al. 2025; Lawson and Hofmann 2026). Additionally epidermal properties such as thickness may also play a role (Drake et al. 2019) which may influence the extent of the mechanical advantage and the GC turgor pressures needed to change aperture (Pichaco et al. 2024; Woning et al. 2026).

## Guard and subsidiary cell morphology and cell wall properties

The conclusions drawn by Franks and Farquhar (2007) regarding the importance of biomechanics for stomatal function have inspired many studies into the mechanisms, ranging from the role of cell wall biochemical composition in guard and SC shape changes to differences in the mechanics of kidney vs dumbbell-shaped GCs.

Generally, GC deformation, as a result of turgor changes, is essential to opening and closing of stomata and is reliant on the structural framework of the plant cell wall (Jones et al. 2003), the biochemical composition of which causes differential thickening that enables repeated swelling and deflation of the GCs. The requirement of anisotropy for this process has long been known; however, our understanding of how this is facilitated by cell wall composition has only recently been improved (Amsbury et al. 2016). A biomechanical model of a GC pair of kidney-shaped stomata illustrated that GC wall microfibers, consisting of cellulose microfibrils with embedded pectin which form fibers around the circumference of GCs, were essential for stomatal opening (Woolfenden et al. 2017). Specifically, increasing turgor in the GCs causes the pectinaceous matrix to stiffen, resulting in the necessary hoop rigidity to create the anisotropy required for opening.

However, Carter et al. (2017) showed that, despite this radial thickening not being present in immature GCs, these cells still display a normal opening response. Using finite element modeling in conjunction with experimental data, the authors demonstrated that radial thickening was not essential for stomatal opening; however, polar stiffening was. This process provides a mechanical explanation of how kidney-shaped stomata function, suggesting pectin-based “pinning down” of GC poles, which limits GC lengths during opening and makes stomatal responses more sensitive to turgor pressure changes, resulting in opening at a lower pressure. The authors showed that the presence of pectin in the cell wall structure in these polar regions may be responsible for this localized stiffness.

The importance of pectin in GC walls to confer differential rigidity essential for stomatal opening has been extensively studied and was recently revised (Koiwa et al. 2026). While plant cell walls are composed mainly of polysaccharides, predominantly consisting of cellulose microfibrils bound by hemicellulose polymers, pectins generally make up 30% of cell walls and are particularly common in GCs. These are branched or linear, and homogalacturonan (a main component of linear pectins) is linked to cell wall rigidity, while branched molecules often contain arabinan, which is associated with greater flexibility (Jones et al. 2003). Low amounts of cellulose and hemicellulose had different impacts on stomatal pore area and degree of opening, while reductions in overall pectin molecular mass changed anisotropy and increased stomatal opening in Arabidopsis, in a study by Yi et al. (2018). The authors discuss the impact of different combinations of polysaccharides on cell wall structure and mechanics of GCs. Interestingly, Rui and Anderson (2016) demonstrated dynamic changes in the rates of cellulose synthesis in Arabidopsis GC walls, with increasing cellulose production during stomatal closure and reorganization of cellulose microfibrils from uniform to bundled distribution, indicating the presence of short-term adjustments of cell wall components during stomatal aperture changes.

Carter et al. (2017) showed that polar stiffening in GCs, as described above, was markedly reduced when treated with polygalacturonase, a cell wall–modifying enzyme that hydrolyzes bonds in the pectin's polygalacturonan matrix, and basal stomatal aperture was significantly lower, confirming the importance of the cell wall pectin network in stomatal function. Similarly, Jones et al. (2003) showed that the breakdown of arabinan is involved in stopping either stomatal opening or closing, a process that can subsequently be reversed by removal of homogalacturonan. The authors also concluded that cell wall–degrading enzymes, in this case arabinans, play an essential role in GC function, with arabinan implicated as a major player in maintaining flexibility of GC walls, opposing the rigidity linked to galacturonan.

The essential function of arabinans in stomatal opening was confirmed by Carroll et al. (2022), who specifically demonstrated the importance of a particular short-chain linear arabinan epitope, associated with rhamnogalacturonan I, the presence of which in GC walls was required for full opening. Interestingly, in transgenic Arabidopsis plants overexpressing arabinan synthase, an increase in longer-chain arabinan epitopes in GC walls was observed, which was associated with a higher maximum stomatal pore aperture due to greater cell wall flexibility under turgor, leading to a larger rounder pore. Authors showed that these plants had higher *g_s_* values at ambient [CO_2_] and greater assimilation rates and iWUE at ambient and elevated [CO_2_]. They concluded that manipulation of GC wall flexibility via arabinan side chains of pectin molecules could provide a target for improved pore aperture, gas exchange, and photosynthesis, notwithstanding the potential impact of other pectin modifications (eg demethylation) on wall flexibility. The importance of pectin methylesterase gene, PME6, to stomatal function was reported by Amsbury et al. (2016), who showed that PME6-1 mutant GCs with walls that contain a greater amount of methyl-esterified pectin had a decreased range of response to environmental opening/closing stimuli, as well as ABA, suggesting that changes in pectin methyl-esterification of GCs mechanically limit the range of pore sizes that can be achieved. While these plants had increased *g_s_*, and hence greater evaporative cooling, they had decreased growth under ambient [CO_2_], as changes in GC-specific cell wall gene expression altered the mechanical properties of GCs limiting the dynamics of stomatal opening and impacting on the ability of the leaf to control water loss.

Investigating changes in the geometry of the four-cell stomatal complex in grasses, ie two dumbbell-shaped GCs with 2 lateral SCs, Durney et al. (2023) showed the importance of the mechanical properties conferred by this specific cellular geometry to stomatal function. These authors determined the significance of cell wall thickening in dumbbell-shaped GCs relative to the contribution of SCs to turgor pressure changes using a finite element model (FEM) and concluded that, while SCs were not essential to achieving some degree of stomatal function, the reciprocal pressure system between GCs and SCs greatly enhanced stomatal responsiveness and complete closure of the aperture could not be realized without the presence of SCs, indicating a direct mechanical role for these cells acting as “springs” against GC lateral movement. Interestingly, and unlike kidney-shaped GCs, wall anisotropy was not necessary for stomatal opening, but thickening of the GC rod region was required for improved pore opening of these dumbbell-shaped GCs. Durney et al. (2023) conclude that the evolutionary success of major crop species may have relied on procurement of these specific cell shapes. Recently, McAdam et al. (2026) have shown that the geometry of the stomatal complex across the order Poales has a major influence on the extent of mechanical interactions between GC and SCs. In grasses, with comparatively large SCs compared to GCs there is considerable mechanical interactions between the two cells, with the GC opening the pore by considerable displacement of the neighboring, paracytic SCs. Consequently, wrong-way responses are considerable in grasses when leaf water status abruptly changes. In contrast to grasses, non-grass species across the order Poales have considerably smaller SCs relative to GCs—because GCs are largely kidney-shaped and the SCs are small in size. This reduced area of mobile epidermis into which stomata open in non-grass Poales greatly reduces mechanical interactions in the four-cell stomatal complexes, resulting in minimal to no wrong-way stomatal responses when leaf water status changes. This work confirms the hypotheses of Franks and Farquhar (2007) SC and GC shape play a major role in determining GC mechanical regulation.

Given the importance of mechanical interactions between GCs and SCs for stomatal function, cell wall properties of SCs may be of equal importance to the biochemical cell wall composition of GCs (Gray et al. 2020), as differences result in different elastic forces of the two cell types (Sharpe et al. 1987). In maize, GC walls are relatively enriched with lignin while SC walls were more pectinaceous (Gallagher and Smith 2000), implying greater flexibility. In a study of how stomatal complex structure impacts on the mechanical advantage of the SCs of the tropical species *Fragraea ceilanica*, Pautov et al. (2024) used 3D modeling to show that the mechanical advantage is reduced due to uneven thickening and rigidity of SC walls, as well as substomatal chambers SCs can expand into when turgor pressure increases, following the earlier work of Franks and Farquhar (2007). Authors conclude that the impact of mechanical advantage greatly depends on stomatal complex structure, which, in the case of *F. ceilanica* allows stomata to open when water status of the leaf is high, conferring a potential adaptive advantage in tropical regions.

The key features of models of stomatal biomechanics, ie cell wall mechanical properties, GC shape and the role of mechanical advantage and surrounding epidermal/SCs have recently been reviewed by Tan et al. (2025), who also discuss the validity of assumptions made in past and current models. Mechanical property modeling greatly relies on mathematical concepts such as Young's modulus and Poisson's ratio; however, plant tissues do not strictly “follow the rules” of these models. Anisotropy or “direction-dependent stiffness” due to cellulose microfibril arrangements in the cell walls of GCs provides one example of such deviation from model approximations. The authors conclude that the challenge remains to combine the biomechanical elements and their interactions with ion flux-based models to provide fully integrative understanding of how stomata work.

## What do mutants tell us about subsidiary cells, guard cells, and the see-saw model?

In maize and other grasses, SCs are formed by asymmetric divisions, and commonly used as a model system to understand the developmental and cellular mechanisms of asymmetric divisions (Harman and Muroyama 2023; Zhang et al. 2023; Berg et al. 2026). Developmental mutants have helped us gain insight into how SCs function, and test some of the predictions made by the see-saw model put forward by Franks and Farquhar (2007)—with important caveats discussed below. Early mutants in SC development include the *brick* (*brk)* and *pangloss (pan)* mutants in maize (Gallagher and Smith 2000). In these mutants, the asymmetric divisions that create SCs are abnormal, and SCs do not differentiate—although this only happens in ∼30% of the divisions. Therefore, in these mutants, there are stomatal complexes with 0, 1, or 2 normal SCs, and normal GCs. Gallagher and Smith (2000) found that when subsidiary mother cells fail to divide properly, daughters adjacent to the GC do not accumulate potassium when the stomatal pore is closed. This implies that as part of their differentiation, SCs express specific genes that promote accumulation of solutes such as potassium; and that simply being adjacent to a GC is not sufficient for potassium accumulation. This is consistent with the Franks and Farquhar “see-saw” model, and with subsequent physiological observations that maize SCs have electrophysiological properties distinct from GCs or pavement cells (Mumm et al. 2011).

More detailed analyses of stomatal function of *pan* mutants lend support for the see-saw model, and the idea that SCs play an active role in the regulation of stomatal aperture. Gas exchange analyses of maize *pan1* and *pan2* mutants indicate that SCs are required for correct stomatal closure (Liu et al. 2024) and wrong-way responses to leaf excision (McAdam et al. 2026 ). By analyzing stomatal apertures in individual stomata with 0, 1, or 2 abnormal SCs, Liu et al. (2024) determined that stomata with abnormal/missing SCs have increased apertures in response to ABA. Notably, in closed stomata with one normal and one abnormal SC, the GCs became curved away from the normal cell, implying a positive or pushing force from the normal SC. Although *pan1* and *pan2* are both defective in the divisions that generate SCs, *PAN2* (and not *PAN1*) is important for stomatal closure when SCs that have divided normally. Interestingly, *PAN2* is orthologous to the Arabidopsis *GC HYDROGEN PEROXIDE RESISTANT 1 (GHR1).* In Arabidopsis, GHR1 regulates the GC-specific anion channel SLAC1, and Arabidopsis *ghr1* mutants do not close their stomata normally (Hua et al. 2012; Sierla et al. 2018). This implies that PAN2 has dual roles: in early stomatal development, and later in stomatal regulation.

In Brachypodium, *pan1* and *polar*, which have similar phenotypes to maize *pan* mutants, also have altered stomatal dynamics as measured by gas exchange (Zhang et al. 2022). Notably, the *Bdpan1* and *Bdpolar* mutants have lower steady state *g_s_,* which is attributable to a slightly smaller stomatal length. This highlights an important caveat when interpreting physiological function using developmental mutants. There can often be developmental (or physiological) compensatory effects, therefore having a robust understanding of mutant phenotypes is essential when using them as tools. Maize *Zmpan1* and *pan2* mutants also have shorter stomatal lengths (Liu et al. 2024). However, while *Zmpan1* has a lower steady state *g_s_*, *pan2* has a higher steady state *g_s_*, likely due to the additional role *pan2* plays in promoting stomatal closure. Having highlighted the caveats of developmental mutants, they have been also extremely beneficial in advancing our understanding of guard and SC interactions and the impact on stomatal function, and without these the field would be reliant on comparative approaches, that do not control for other differences beyond stomata, ie photosynthetic type and capacity.

The ideal mutant to analyze SC function would have no SCs, but normal GCs. In Brachypodium, the mutant *SC identity defective (sid),* lacks SCs (Raissig et al. 2017). Cloning of the gene revealed that *SID* was encoded by the transcription factor *BdMUTE*. In Arabidopsis, *AtMUTE* is expressed in GC precursors, and is important for GC development (Pillitteri et al. 2007). *BdMUTE* is transcribed and translated in GCs and then moves to adjacent cells to induce SC fate. Consistent with this expression pattern, *Bdmute* has GC defects in addition to SC defects, albeit at a much lower frequency than the SC defects (Spiegelhalder et al. 2024). Raissig et al. (2017) found that *Bdmute* had an overall lower rate of change in *g_s_* and prolonged stomatal closure times. To try and disentangle the subsidiary and GC defects, Spiegelhalder et al. (2024) used a non-motile form of BdMUTE to rescue the mutant phenotype, such that MUTE would be in GCs, but not SC precursors. Notably, the presence of SCs influenced the shape of the GCs, suggesting that biomechanics might influence GC development. Gas exchange analyses of lines with different frequencies of normal SCs and GCs reinforced the idea that SCs are important for stomatal dynamics.

In maize, *Zmmute*, was identified as *bizui2 (bzu2)* in a screen for mutants with elevated leaf temperature (Wang et al. 2019a). Plants with high leaf temperature typically have lower evaporative cooling due to closed stomata. Like *Bdmute, Zmmute* fails to make SCs. However, *Zmmute* has a more severe GC defect than *Bdmute*, and is seedling-lethal (Wang et al. 2019a). The maize *lost SC (lsc)* was isolated in a similar screen for mutants with elevated leaf temperature (Cui et al. 2023). Abnormal stomatal complexes in the *lsc* mutant are striking. About half of the stomatal complexes lack SCs, but often the shape of the cell seems to be reminiscent of a SC, unlike in *pan* or *mute* mutants (Cui et al. 2023). *LSC* encodes a ribonucleotide reductase—how this leads to abnormal SCs is unclear but may be related to cell cycle regulation. The *lsc* mutant has clear defects in stomatal conductance, but no documented effects on the GCs themselves. A more detailed analysis of the anatomy and function of the stomatal complex may help us understand how SCs contribute to stomatal function.

Franks and Farquhar compared GCs with and without SCs, and they compared kidney- vs dumbbell-shaped stomata. Before maturation, grass stomata start as kidney-shaped and attain their characteristic dumbbell shape through cell wall thickening. It seems plausible that grass mutants with kidney-shaped GCs should be obtainable. The *bizui3 (bzu3)* mutant was isolated in the same screen as *bzu2/Zmmute* (Zhou et al. 2023). BZU3 encodes an epimerase that is important for cell wall biosynthesis, and results in GCs that lack their characteristic dumbbell shape. Instead, the GCs have “rod” shapes that are neither kidney nor dumbbell-shaped, or thin “antenna” shapes. These mutants have severe stomatal defects and are seedling-lethal because they abort before maturation.

Thus far, the majority of the characterized SC mutants have been developmental mutants, where SCs are not specified and/or formed correctly. A notable exception is *closed stomata 1* (*cst1*) (Wang et al. 2019b). *CST1* encodes a member of the SWEET family of glucose transporters. The *cst1* mutant has lower stomatal conductance (*g_s_*), at the onset of flowering and an early senescence phenotype caused by carbon starvation due to decreased stomatal apertures. CST1 is specifically expressed in SCs, and its expression is negatively regulated by photo-assimilates. The working model for CST1 is that it is a positive regulator of stomatal opening, and when the plant is sugar replete, this decreases CST1 expression, leading to stomatal closure. Importantly, it suggests that the role of SCs includes regulation of stomatal function.

## What have mutants told us about subsidiary cell function in non-grass models?

Unfortunately, there are relatively few non-grass genetic models that also have anatomically distinct SCs. *Kalanchoe laxiflora* is a CAM dicot in the Crassulaceae with distinct SCs (Cheng et al. 2026). Unlike in the grasses, where SCs are recruited from pavement cells adjacent to the cell that eventually forms GCs (perigenous stomata), the SCs in *K. laxiflora* are derived from the same cell lineage as the GC progenitor. *K. laxiflora* typically has three SCs (anisocytic) rather than paired SCs (paracytic), which makes the mechanical relationship between the guard and SCs in *K. laxiflora* distinct. However, similar to grasses, *K. laxiflora* SCs accumulate potassium when stomata are closed, suggesting a supportive role (Cheng et al. 2026).

The Franks and Farquhar see-saw model predicts that there are reciprocal fluxes of solutes between subsidiary and GCs. This raises the question: are the channels and transporters that move solutes across membranes identical in guard and SCs, and oppositely regulated; or do the different cell types have distinct (although likely related) proteins? How do SCs and GCs coordinate their functions? Note that the answers to these questions may not be the same in different species, given that it is likely that SCs evolved multiple times. RT-PCR of individual cells indicates that maize guard and SCs have both shared and distinct shaker-family potassium channels (Büchsenschütz et al. 2005). Unfortunately, mutants in ZMK1, a channel expressed in SCs but not GCs, are embryo lethal (Philippar et al. 2006). Single cell RNA-seq (scRNA-seq) or single nuclear RNA-seq (snRNA-seq) datasets will help address these questions. Importantly, correct identification of mature subsidiary and mature GCs after sequencing is essential, and there are relatively few reliable SC marker genes other than *CST1.* An added complexity is that mature grass GCs have heavily lignified cell walls and therefore can be underrepresented in scRNA-seq or snRNA-seq datasets. Existing datasets such as the maize dataset produced by Sun et al. (2022) and the promoter set by Nguyen et al. (2025) and the recently published single cell atlas from grass epidermes by Berg et al. (2026) which will help identify subsidiary vs GC-specific transporters and regulators. Of particular interest would be comparative datasets across multiple species with and without SCs.

## Conclusion

The visionary and pioneering work of Franks and Farquhar (2007) stands out in stomatal biology. It has taken nearly two decades for new methods to be developed and utilized to provide additional data on GC and epidermal cell turgor across species (Yi et al. 2022; Brodersen et al. 2025), and to visualize mechanical interactions between GCs and epidermal pavement cells when stomata open and close (Manandhar et al. 2026). The work of Franks and Farquhar (2007) continues to provide a number of inspiring questions about stomatal mechanics and evolution, as well as the importance of cell wall structure in both GCs and SCs, and the interaction and ion fluxes between the two cell types and the impact these have on stomatal function (including the rapidity of responses).

Advances box

- The role of SCs is as important as GCs in regulating stomatal kinetics, contributing both physically and biochemically.
- The mechanical advantage of the SCs over the GCs has been important in the evolution of stomata to support more rapid kinetics and increased photosynthetic capacity.
- SCs exhibit substantial diversity in their structure, number, and function, yet their precise mechanistic contribution to stomatal behavior remains poorly resolved.
- Stomatal aperture is regulated by both hydroactive and hydropassive mechanisms, although the extent of these may differ between species and plant types.
- Despite its importance, maximum GC turgor pressure in open stomata has been quantified for only a limited number of species.

Outstanding questions

- How do SCs regulate stomatal function?
- How does variation in SC size and shape influence stomatal behavior?
- Which developmental and environmental factors shape the diversity of stomatal complex architectures, and in what ways do these structural variations affect stomatal function?
- Can we obtain grass mutants with kidney-shaped GCs that are nonetheless flanked by SCs?
- Are the channels and transporters that move solutes across membranes identical in GCs and SCs?
- How do SCs coordinate with GCs?
- How do SCs interact with surrounding epidermal cells?
- Can we manipulate cell wall properties in SCs to alter stomatal behavior?
- In species without SCs, do the adjacent epidermal cells play a similar role?

## Data Availability

There are no new data associated with this article.
